# Differentiated antiretroviral distribution: Implementation in five South African districts

**DOI:** 10.4102/phcfm.v17i1.4974

**Published:** 2025-08-27

**Authors:** Justin Engelbrecht, Chandbi Tajeer, Cara O’Connor, Kate Rees

**Affiliations:** 1 Anova Health Institute, Johannesburg, South Africa; 2 Department of Community Health, Faculty of Health Sciences, University of the Witwatersrand, Johannesburg, South Africa

**Keywords:** differentiated service delivery, antiretroviral therapy, differentiated models of care, HIV, CCMDD

## Abstract

**Background:**

The National Department of Health introduced Differentiated Service Delivery (DSD) models to improve retention in care and decongest healthcare facilities. Anova Health Institute supported the implementation of DSD guidelines in five districts of South Africa.

**Aim:**

The study aimed to describe how the models contained in DSD policies are operationalised.

**Setting:**

Five districts of South Africa – two metropolitan, two mixed and one rural.

**Methods:**

We used a mixed-methods approach, incorporating a 2-day participatory workshop in 2023 and a retrospective review of routine programmatic data. A mapping exercise was used to understand all models of chronic medication provision in the five study districts and to describe differences in operationalisation. We also report on the number of options per facility and healthcare provider perspectives of benefits and limitations.

**Results:**

External and facility pick-up points were the most commonly implemented models. Three key themes were: the trade-off between convenience and additional support, the trade-off between controlling client care and outsourcing tasks and the distribution of work between cadres of staff. Sedibeng District provided the most options per facility, with 57% of facilities having three possible options. Cape Town provided the fewest, with 50% of facilities offering only one option.

**Conclusion:**

Health and environmental contexts guide the choice of DSD modalities offered. It is possible to offer clients options in South African settings.

**Contribution:**

This study highlights the context-specific nature of DSD model implementation and the importance of client choice. Further research into availability and options from a client perspective would be useful.

## Background and introduction

South Africa has a complex disease burden, including a high prevalence of human immunodeficiency virus (HIV), with approximately 7.4 million people living with HIV and 5.5m on antiretroviral therapy (ART).^1^ As countries strive towards achieving Joint United Nations Programme on HIV and AIDS (UNAIDS) 95-95-95 targets, there is an increased focus on models of Differentiated Service Delivery (DSD), which facilitate available and accessible health services.^2^

In the South African context, DSD refers to delivery of healthcare services that differ from standard models of care in terms of the type of service, location, which individuals receive the service, and when the service is provided.^3^ For example, DSD models for medication provision include E-lockers, home deliveries, adherence clubs and vending machines, among others.

When used to provide chronic medication, including ART, DSD models have been shown to increase access to medication, decongest healthcare facilities and reduce waiting times. These improvements contribute to reduced missed appointments, improved retention and improved viral load suppression.^4,5,6^ A key value of DSD models is their flexibility and client-centredness, placing clients at the centre of the service design, empowering them to self-manage their health and, ideally, providing clients with a choice as to which service model to use.^7^

In South Africa, DSD is guided by the *Adherence Guidelines for HIV, Tuberculosis and non-communicable diseases (NCDs)*, developed by the National Department of Health in 2016 and updated in 2020. These guidelines encourage and support the implementation of DSD models to make it easier for people with chronic illnesses to access treatment and stay in care.^7^ They include Standard Operating Procedures (SOPs) for specific DSD modalities for chronic medication provision. All Department of Health facilities are encouraged to adopt context-specific models to ensure clients have access to their medication. The DSD modalities described in the SOPs are facility pick-up points (medication ‘fast lanes’), adherence clubs and external pick-up points.^7^

With a supportive policy framework in place, DSD models have been widely implemented across South Africa. The Centralised Chronic Medicines Dispensing and Distribution (CCMDD) programme was established in 2014. Through the CCMDD programme, the National Department of Health contracts with private service providers to pre-pack, distribute, dispense and collect undispensed medication at designated pick-up points, including external pick-up points such as community-based outreach or adherence club locations, independent pharmacies and private medical practices. This public–private partnership has been essential for the expansion of chronic disease programmes and support for people living with HIV and other chronic conditions. The CCMDD programme has a robust recording, data and electronic management system, known as Synchronised National Communication in Health (SyNCH), which enables electronic prescriptions, automated reporting and promotes rational prescribing and compliance with the Standard Treatment Guidelines (STGs).^8^ The CCMDD programme has a set formulary that includes medications for HIV, non-communicable diseases (e.g. diabetes and hypertension) and other health needs (e.g. oral contraceptives). This integration of HIV services with NCD care helps optimise resources use and address comorbidities in tandem.^8^

Despite its reach, CCMDD is not available to all clients. Some may be excluded due to clinical eligibility criteria, a lack of identification documents required for SyNCH registration, and geographic availability (CCMDD is not available in Western Cape province). As an alternative to CCMDD, the Department of Health (DOH) runs chronic dispensing units (CDU). The Western Cape operates a CDU model similar to that of other provinces, implemented since 2005, but delivered as an outsourced, public sector centralised dispensing service.^9^

Beyond CCMDD and CDU, a variety of DSD modalities have been introduced or are in development. In 2023, the Western Cape province published an updated provincial framework for Differentiated Models of Care (DMOC) outlining 13 potential modalities for medication collection.^3^ This framework builds on existing models and is designed to be flexible and non-prescriptive.^3^

As the CCMDD and DMOC programmes have strengthened alternative distribution and access to medicine, other role-players, such as community-based organisations, civil society groups and private sector partners, including participating retailers, play a vital role in expanding medication access. These actors provide financial, infrastructural and logistical support. Non-profit organisations have been key partners to the DOH in implementing the HIV response, and have played an important role in innovating, testing and scaling up DSD services. For example, Right to Care has been instrumental in the developing E-lockers and ATM-style medication vending machines. The Anova Health Institute, a non-profit organisation, previously funded by the United States President’s Emergency Plan for AIDS Relief (PEPFAR) through the United States Agency for International Development (USAID), supported the Department of Health to implement and monitor HIV and tuberculosis (TB) programmes, including DSD models, across five South African districts. The Anova nurses, pharmacists and other staff worked alongside DOH in facilities to implement DSD and collected, analysed and reported programme data to stakeholders.

While several studies have described the current CCMDD model, there is limited evidence of how the broader complex array of DSD modalities has been implemented at facility level.^9,10,11^ This study aims to describe how the DSD modalities contained in the National Adherence Guidelines and Western Cape Government DMOC Framework have been operationalised by the Department of Health, with support from the Anova Health Institute.

## Methods

### Setting

The study was conducted in public health facilities in five districts in South Africa: City of Johannesburg and Sedibeng districts in Gauteng province; Capricorn and Mopani districts in Limpopo province; and City of Cape Town in the Western Cape province. Johannesburg and Cape Town are urban metropolitan districts, Sedibeng and Capricorn are peri-urban or mixed, while Mopani is largely rural.

### Study design and recruitment

The analysis used a mixed-methods approach, incorporating a retrospective review of routine programmatic data and a participatory workshop. To understand contextual factors affecting implementation of the DSD programme, a 2-day participatory workshop was held in July 2023 in Gauteng province and attended by the Anova managers responsible for DSD, from each of the five districts. Participants were selected purposively as they were responsible for providing technical and health system strengthening support for DSD programmes, making them familiar with the daily activities of facility staff as well as implementation challenges and best practices.

Participants conducted a mapping exercise to identify all models of chronic medication provision in each district, describing the models and the differences in operationalisation. A World Café style engagement^12^ was facilitated by the Anova’s Public Health Medicine Specialist to understand how participants perceived five common modalities (adherence clubs, e-Lockers, external pick-up points, facility pick-up points and quick pick-up points in trailers). We limited the number of modalities discussed to five because of time constraints and a desire to explore the chosen modalities in detail. We selected the commonest modalities prioritised for expansion across the districts (facility pick-up point; external pick-up point; adherence clubs and e-Lockers), as well as quick pick-up points, as they have not been previously described. The public health specialist explained the purpose of the 2-day workshop and how data gathered would be used in the final write-up of this article. Participants were asked to consider the advantages and disadvantages of each model from a client, pharmacy and clinician perspective. There were five World Café tables, one for each model.

We captured outputs from the World Café exercise into a shared spreadsheet, using free text. This allowed for some standardisation in terms of questions answered (e.g. advantages, disadvantages from the point of view of patients, pharmacists and clinicians), and flexibility to capture discussions. Two researchers (public health specialist and public health analyst) kept field notes to complement the spreadsheet. We did not record the proceedings.

### Data analysis

A thematic analysis of the outputs was conducted to extract and collate the most impactful and frequently occurring advantages and disadvantages of the various models. Participants in this study were requested to consider their responses from a patient, pharmacist and clinician point of view. The data were organised into codes which were then arranged into themes, under the overarching categories: benefits and limitations. There was no framework used, and coding was purely inductive.

For the retrospective review of routine programme data, investigators collected the following from aggregated programme reports: number of facilities per district implementing DSD, the type(s) of DSD per facility and the number of patients on ART per facility. Data were extracted for September 2023. No programmatic outcomes were analysed.

### Ethical considerations

This study was approved by the Human Sciences Research Council Health Research Ethics Committee (reference REC 3/22/08/18). Informed consent was not required for participatory workshop participants or the use of aggregated data. All procedures performed in these studies involving human participants were in accordance with the ethical standards of the institutional and/or national research committee and with the 1964 Helsinki Declaration and its later amendments or comparable ethical standards.

## Results

We describe DSD types for 601 health facilities across 5 districts. Eighteen healthcare managers from five districts participated in the mapping workshop and World Café session.

### Description of differentiated service delivery models

Workshop participants described the various DSD models and how they were operationalised. We found that across the 5 districts, 10 modalities were being implemented. In the Western Cape policy, reference is made to a selection tool when comparing modalities and/or prioritising multiple modalities. A scoring system was used to address questions, such as whether the model addressed a service need or a barrier to care in the community.^3^ At national level, models should be selected based on evidence based interventions to support retention in care.^13^

### Implementation of differentiated service delivery models

The number of facilities providing each DSD model per district is described in Table 1. We also indicate whether the client needed to enter the facility for each model, which has implications for convenience and decongestion. Additional comments highlight where models had differed substantially across districts. The most widely used model was facility-pick-up points, available in all five districts and at 377 facilities (63% of all facilities), followed by the external pick-up points, available in all five districts and 344 facilities (57%). Five modalities were provided in fewer than 10 facilities: community adherence clubs, quick pick-up points, outreach, wellness hubs and home delivery.

**TABLE 1:** Differentiated service delivery models by district in the five Anova-supported districts and facilities in September 2023.

DSD Model	Number of districts implementing		Number of facilities implementing (*N* = 601)		Number of clients on ART at a facility with this model (*N* = 593 420)		Definition	Characteristics							
	*n*	%	*n*	%	*n*	%		Autogenerated SMS reminders	Community settings	Flexible collection time	Identity documents required	Mobile phone required	Other health services available	Self service	Support and counselling provided
Facility pick-up point	5	100	377	63.0	409 291	69.0	Clients pick up pre-packed medication at a facility either at the pharmacy or designated rooms. Can be through CCMDD or not.	-	-	X	-	-	X	-	-
External pick-up points	5	100	344	57.0	420 895	70.0	Clients receive medication through CCMDD. Medication is distributed by an external service provider to a pick-up point in the community, for example, independent pharmacies, private general medical practices, and other accredited venues.	X	X	X	X	-	-	-	-
Adherence club: Facility	4	80	164	27.0	113 669	19.0	Group of up to 30 clients meet every 2 to 3 months, at a facility. Facilitated by a counsellor who issues pre-packed medicine and completes registration.	-	-	-	-	-	X	-	X
Adherence club: Community	1	20	5	0.8	905	0.1	Similar to Facility Adherence Clubs above, but clients meet at an off-site area such as community halls.		X	-	-	-	-	-	X
E-lockers	2	40	45	7.0	90 891	15.0	Pre-packed medication is delivered to lockers. Lockers are opened by the client using a one-time password. They may be located at facilities or at community locations such as shopping malls. Can be serviced by external service providers or pharmacists.	X	X	X	X	X	-	X	-
Fast-Lane	1	20	16	3.0	9501	2.0	Non-grouped clients collect their medicine at a pharmacy window on a specific day.	-	-	X	-	-	X	-	-
Quick pick-up points	1	20	6	1.0	3001	0.5	Grouped clients collect their medicine during a specific time slot either at a pharmacy window or at a trailer.	-	X	-	-	-	X	-	-
Outreach	1	20	39	1.0	65 362	11.0	Similar to external pick-up points, although facility-nurse-driven and could include clinical services.	-	X	-	-	-	X	-	-
Wellness hub	1	25	2	0.3	2501	0.4	Spaces such as community halls or churches that offer a variety of services, including blood pressure checks and pre-packed medication collection.	-	X	-	-	-	X	-	-
Home delivery	2	40	7	1.0	4001	0.6	Used during the COVID-19 era to allow vulnerable clients to receive medicines without visiting facilities. Pre-packed medicines were prepared by an external service provider and delivered to patients’ homes *via* private delivery services or community health workers.	-	X	-	-	-		-	-

DSD, differentiated service delivery; ART, antiretroviral therapy; CCMDD, centralised chronic medicines dispensing and distribution; COVID-19, coronavirus disease 2019.

### Distribution of differentiated service delivery models

**FIGURE 1:**
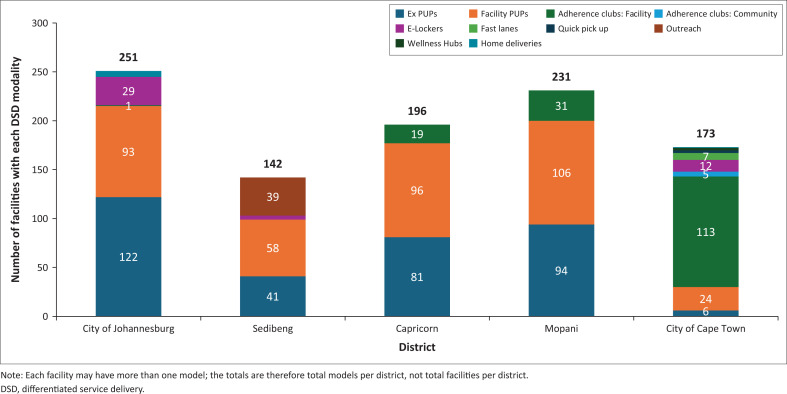
Number of facilities offering each differentiated service delivery model by district in September 2023.

Figure 1 represents the number of DSD models available within each of the Anova-supported districts. The patterns of availability of the different models varied across the districts. There were five DSD models available in only one district, reflecting adaptations to address local needs (namely, quick pick-up points, fast lanes, wellness hub, home delivery and internal CDU). City of Johannesburg facilities had more external pick-up points (*n* = 122, 100%) and facility pick-up points (*n* = 93, 76%); Mopani had more facility pick-up points (*n* = 106, 77%); City of Cape Town had more adherence clubs (*n* = 113, 82%); and Sedibeng had more outreach (*n* = 39, 67%). There were two wellness hubs and one home-delivery service in City of Cape Town and City of Johannesburg, supported through community health workers. Capricorn and Mopani, both rural areas, had fewer options overall due to geographical and infrastructural contexts with less availability of private providers.

Table 2 presents the number of potential options available to clients per facility in each of the five districts. On average, City of Johannesburg had the highest number of modalities per facility, at two, with Mopani the lowest at one. Moreover, 57% of Sedibeng facilities offered three modalities, while 68% of City of Johannesburg, 42% of Capricorn, and 47% of Mopani facilities offered two modalities. The total number of clients on ART at facilities with more than one option was 577 415, 59% of 972 481 active ART clients.

**TABLE 2:** Number of differentiated service delivery modalities per facility in September 2023.

Number of facilities with DSD options among five districts	Number of facilities with DSD options									
	0 models/options		1 model/option		2 models/options		3 models/options		4 models/options	
	*n*	%	*n*	%	*n*	%	*n*	%	*n*	%
City of Johannesburg (*n* = 122)	9	7	19	16	83	68	20	16.0	0	0
Sedibeng (*n* = 58)	0	0	13	22	9	16	33	57.0	3	5
Capricorn (*n* = 145)	46	32	20	14	61	42	1	0.7	0	0
Mopani (*n* = 138)	29	21	14	10	65	47	29	21.0	0	0
City of Cape Town (*n* = 138)	31	22	69	50	23	17	7	5.0	0	0

DSD, differentiated service delivery.

### Participant perception of modalities

Table 3 summarises the benefits and limitations of the most widely used modalities, as discussed by the participants. There was no one preferred model, rather with the participants recognising advantages and disadvantages of each in terms of access potential, benefits for healthcare workers and feasibility across different contexts. All modalities were regarded as beneficial overall, particularly in helping to decongest busy clinic and pharmacy spaces, but participants highlighted that the administrative burden of managing them remains substantial.

**TABLE 3:** Summary of differentiated models of care model benefits and limitations from the perspective of healthcare managers.

DSD models	Benefits	Limitations
Adherence clubs (facility or community)	Non-clinical staff can facilitate	Administrative burden for planning and preparation
	Other health services available	Dependent on clinic hours
	Perception of more control in clinical management	Facilities need to provide the human resources
	Scripting calendar assists with planning and identifying viral loads due	Longer waiting times for high volume clubs (> 30 clients)
	Sometimes available in community settings	Space constraints
	Support and counselling integrated into model	-
E-lockers	Autogenerated SMS reminders	Administration burden registering and troubleshooting (e.g. lost pin)
	Facility decongestion	Difficult for facility to identify clients who missed pick-ups
	Flexible collecting hours	Less interaction with facility staff
	Minimal impact on clinical team	Loadshedding and network issues
	Pharmacy decongestion/less stock to manage	Long registration process
	Quick service	Mobile phone requirement
	Self-service	Potential for theft and damage
	Sometimes available in community settings	Script rejection needs facility visit to rectify
External pick-up points and outreach	Autogenerated SMS reminders	Administrative burden registering and managing unscheduled returns
	Available in community settings	Difficult for facility to identify clients who missed pick-ups
	Facility decongestion	Late deliveries to pick-up points
	Flexible collecting hours	Less interaction with facility staff
	Minimal impact on clinical team	Long registration process
	Minimise stigma	Script rejection needs facility visit to rectify
	Pharmacy decongestion/less stock to manage	-
	Quick service	-
Facility pick-up points and fast lane	Flexible collecting hours	Dependent on clinic hours
	No identity documents needed	Less interaction with facility staff
	Other health services available	Space constraints
	Perception of more control in clinical management	-
	Quick service	-
Trailers and containers	Burden of loading and offloading parcels	Administrative burden for planning and preparation
	Facility decongestion	Dependent on clinic hours
	No identity documents needed	Less interaction with facility staff
	Non-clinical staff can facilitate	Potential for theft and damage
	Other health services available	Weather impacts access
	Pharmacy decongestion/less stock to manage	-
	Quick service	-
	Scripting calendar assists with planning and identifying viral loads due	-

DSD, differentiated service delivery.

We identified three main themes running through the discussions. Firstly, the trade-off between convenience and quick service *versus* the need for additional support and counselling. It was widely recognised that clients need easy ways to access medication that minimise disruptions to their daily lives. However, there were also concerns raised among the same participants that clients have not received adequate quality counselling and, as a result, might not be able to maintain viral suppression without frequent input from healthcare workers. This issue was particularly apparent for out-of-facility modalities, which were thought to be very advantageous in terms of access, but participants raised concern about the quality of care provided and the limited potential to develop relationships between community-based clients and facility-based healthcare workers.

Secondly, there was a similar trade-off between maintaining control over client care and experiencing the benefits of handing over tasks to external service providers. Although outsourcing some work (e.g. prepacking medication) was felt to be advantageous, there was a prevailing perception that outsourcing led to loss of control over client care, which caused discomfort among participants. One specific issue was identified that impacts client care: facility-based healthcare workers often do not know when clients miss medication collections at out-of-facility modalities, which means they cannot conduct activities to support retention, for example, telephonic reminders and counselling. Resolving this issue would likely help healthcare workers to feel more in control of client care.

Finally, the distribution of work among clinicians, pharmacists and non-clinical staff was crucial and impacted heavily on whether the model was feasible in certain districts or facilities. Many models demand substantial pharmacist or pharmacist assistant input, which greatly limits their feasibility in numerous settings. External service providers mitigated this challenge.

## Discussion

In describing the availability of various DSD models across five South African districts, we found that coverage of DSD services was broad and that, from the perspective of healthcare workers, it is feasible to implement a variety of DSD options within public sector services. These models provide both clients and providers with choice regarding care delivery. Our findings align with those from three recent studies in South Africa showing that a range of DSD modalities can be effectively operated in public-sector settings,^14^ and that offering clients the opportunity to choose a DSD model based on their needs is feasible.^15,16^

In our study, the most common DSD modalities were facility pick-up points located within public healthcare facilities (377 facilities, 63% of facilities) and external pick-up points situated at private pharmacies, general practitioners’ offices and other community settings (344 facilities, 57% of facilities). Overall, out of 972 481 clients active on ART across the five districts, 55% (*n* = 577 415) received services in a facility that offered more than one DSD model, potentially enabling greater choice for stable clients.

We observed substantial variability in the availability of DSD models, driven by both policy and geographic context. While South Africa’s national DSD policy outlines three broad categories of DSD model – adherence clubs, external pick-up points and facility pick-up points^8^ – the Western Cape’s policy document outlines 13 options.^3^ Both the policies are flexible and non-prescriptive, allowing for context to influence the selection and implementation of DSD models. The Western Cape, as the first province to implement DSD models, pioneered the adherence club model. However, because of policy restrictions on cooperation with private pharmacies, it was also the only province not implementing CCMDD.^17^ This has led to a different mix of models evolving in the Western Cape, including quick pick-up point trailers. While adherence clubs were largely replaced by CCMDD, external pick-up points or facility pick-up points in most parts of the country, they remain the most prevalent DSD models both in City of Cape Town and in Mopani district.

Geographic setting was another key factor driving variation. Rural districts in our study offered fewer options for external pick-up points than urban areas, largely because of the limited presence of private pharmacies or general practitioners that were capable of meeting the contractual requirements of CCMDD. To expand access in these settings, it is important to consider alternative distribution points such as farms, shops or traditional health practitioners. Additional support for private providers in rural districts – including donations of equipment such as computers and air conditioners – may also be necessary to meet contracting criteria.

Differentiated Service Delivery models are known to result in at least equivalent outcomes to standard clinic-based care, with additional benefits from the clients’ perspective such as reduced cost.^18^ In addition, there may be benefits for service providers. A study conducted in rural Nigeria showed a reduced workload for healthcare providers, decreased waiting times and lower transport costs for clients, as well as decongestion of facilities.^19^ Similarly, a multi-country study showed increased job satisfaction among facility staff.^20^ However, participants in our study highlighted the significant administrative burden of DSD models, emphasising the need for careful planning and resource allocation to ensure their efficiency.

In our World Café discussion of benefits and limitations, no single model stood out as preferred by participants. Each DSD model presented distinct benefits and challenges for both clients and providers. Facility-based models, such as adherence clubs and facility pick-up points, were seen to decongest healthcare facilities, carry fewer operational barriers and allow more direct interaction between healthcare workers and clients. These findings are consistent with studies from the Cape Winelands, where most health workers reported that adherence clubs reduced their workload and eased congestion.^21^ However, facility-based services were often perceived as less anonymous, potentially exposing clients to stigma. This challenge, also reported in KwaZulu-Natal and Khayelitsha,^14,22^ was compounded by waiting times linked to over-enrolment and rigid medication collection deadlines. Many healthcare workers recommended extending the 5-day grace period for medication pick ups in effect in the Western Cape at the time of the workshop, to improve client flexibility and reduce administrative burden.

External pick-up points were valued for reducing nurse workload, improving client waiting times, decongesting facilities and minimising stigma. Yet, they were also associated with lengthy registration processes and administrative demands. Delays in medicine parcel deliveries, reported in our study and others, created further barriers to effective service delivery.^23,24,25^

E-lockers and trailers offered enhanced privacy and stigma reduction, while also supporting facility decongestion. A 2021 study from Johannesburg found E-lockers outperformed other modalities in client satisfaction and privacy.^25^ Our participants similarly felt that E-lockers, external pick-up points and trailers helped manage stigma. However, unlike other settings where E-lockers reduced staff workload, our study found that enrolling clients in this service created an administrative burden for healthcare workers.^19^

Infrastructure challenges also affected DSD implementation. Trailers and E-lockers located on facility grounds were vulnerable to theft and damage. In rural areas, E-lockers were largely confined to facilities because of limited infrastructure and fewer private providers. As a cloud-based technology requiring mobile phones to access PIN codes, E-lockers were further constrained by unreliable electricity and network availability. These logistical and infrastructural limitations must be considered when planning DSD service expansion.

Our findings highlighted the tension between client-centred priorities (such as convenience and flexibility) and provider concerns around maintaining oversight of medication dispensing, ensuring appropriate clinical management and integrating adherence and psychosocial support within service delivery. There was a general concern that models allowing for clients to collect medication without entering a facility or seeing a counsellor could lead to poorer outcomes. In our study, participants also reported that clients themselves feared having less interaction with facility staff, echoing findings from a 2019 Ugandan study where clients reported concerns about detachment from the health system when using external or facility pick-up points.^26^

Across modalities, our findings illustrate that no single DSD model offers a perfect solution. Each option presents trade-offs between convenience, clinical oversight, stigma management and administrative burden. This reinforces the need for a diverse mix of modalities and client choice, tailored to local context.

### Limitations

This study focused on the perspectives of healthcare workers, and we did not include community or client participants. As a result, we could not assess user preferences or perspectives on the acceptability and feasibility of different DSD models. Client outcomes by DSD model were also not assessed, as this was beyond the scope of this study. Additionally, we did not evaluate the fidelity of DSD model implementation, nor did we measure uptake, scalability or sustained use over time. These are critical aspects of implementation that would require longitudinal and mixed-methods research to explore comprehensively. Finally, the provision of and enrolment in DSD models is dynamic and changes frequently; the figures presented reflect a single point in time (September 2023).

### Recommendations

Evidence shows that integrated service delivery models for HIV treatment improve retention in care, medication adherence and viral load suppression.^27,28,29,30^ Policymakers should prioritise enrolling more eligible clients into DSD models, enhancing the valued characteristics of existing modalities and expanding eligibility criteria to reach a larger proportion of the client population. Successful implementation depends upon a supportive policy environment and a robust, reliable medication supply chain.^31^

This study highlights the context-specific nature of DSD model implementation and the practical feasibility of offering client choice. Although there has been extensive research conducted on DSD models, including the CCMDD model, the variability in implementation across contexts has not been fully documented. Further research on the availability and need for different options from the client perspective would be useful. Further evaluations could also explore the association between DSD options and viral suppression, as well as the impact of client choice on health outcomes.

## Conclusion

This study described the implementation of DSD models within a large-scale national programme, highlighting how health system and environmental contexts influenced the type and number of options available. Full implementation of national and provincial DSD policies has the potential to improve access to chronic medication, reduce healthcare facility congestion and expand client choice – critical steps towards strengthening the South African healthcare system. While external pick-up points are more readily accessible in urban areas, models such as E-lockers may be better suited to rural settings. Wherever possible, implementers should offer clients a mix of DSD options, tailored to their client-specific needs and circumstances.

## References

[1] UNAIDS. Fact sheet 2022. Global HIV & AIDS statistics [homepage on the Internet]. [cited 2025 Jun 30]. Available from: https://www.unaids.org/en/resources/fact-sheet

[2] Grimsrud A, Bygrave H, Doherty M, et al. Reimagining HIV service delivery: The role of differentiated care from prevention to suppression. J Int AIDS Soc. 2016;19(1):21484. 10.7448/IAS.19.1.21484 27914186 PMC5136137

[3] Western Cape Government: Health and Wellness. A framework for the implementation of differentiated models of care Service Design Reform considering the COPC principles Western Cape Government health & wellness. Cape Town: Western Cape Government Health and Wellness.

[4] Alamo ST, Wagner GJ, Ouma J, et al. Strategies for optimizing clinic efficiency in a community-based antiretroviral treatment programme in Uganda. AIDS Behav. 2013;17(1):274–283. 10.1007/s10461-012-0199-9 22610422 PMC3887144

[5] Christ B, van Dijk JH, Nyandoro TY, et al. Availability and experiences of differentiated antiretroviral therapy delivery at HIV care facilities in rural Zimbabwe: A mixed-method study. J Int AIDS Soc. 2022;25(8):e25944. 10.1002/jia2.25944 36008925 PMC9411726

[6] Obua C, Kayiwa J, Waako P, et al. Improving adherence to antiretroviral treatment in Uganda with a low-resource facility-based intervention. Glob Health Action. 2014;7(1):24198. 10.3402/gha.v7.24198 24909408 PMC4049133

[7] Grimsrud A, Wilkinson L, Eshun-Wilson I, Holmes C, Sikazwe I, Katz IT. Understanding engagement in HIV programmes: How health services can adapt to ensure no one is left behind. Curr HIV/AIDS Rep. 2020;17:458–466. 10.1007/s11904-020-00522-1 32844274 PMC7497373

[8] Prust ML, Banda CK, Nyirenda R, et al. Multi-month prescriptions, fast-track refills, and community ART groups: Results from a process evaluation in Malawi on using differentiated models of care to achieve national HIV treatment goals. J Int AIDS Soc. 2017;20(S4):41–50. 10.7448/IAS.20.5.21650 PMC5577715 28770594

[9] Magadzire BP, Marchal B, Ward K. Improving access to medicines through centralised dispensing in the public sector: A case study of the Chronic Dispensing Unit in the Western Cape Province, South Africa. BMC Health Serv Res. 2015;15(1):513. 10.1186/s12913-015-1164-x 26577831 PMC4650275

[10] Liu L, Christie S, Munsamy M, et al. Expansion of a national differentiated service delivery model to support people living with HIV and other chronic conditions in South Africa: A descriptive analysis. BMC Health Serv Res. 2021;21(1):463. 10.1186/s12913-021-06450-z 34001123 PMC8127180

[11] Health Systems Trust. The Synchronised National Communication in Health (SyNCH) system is a seamless way of linking patients to care in the 21st century [homepage on the Internet]. 2023 [cited 2025 Jun 30]. Available from: https://www.hst.org.za/media/blog/Lists/Posts/Post.aspx?ID=174#:~:text=SyNCH%20is%20a%20real-time,rational%20prescribing%20of%20essential%20medicines

[12] Burke C, Sheldon K. Encouraging workplace innovation using the “World Cafe” model. Nurs Manage. 2010;17(7):14–19. 10.7748/nm2010.11.17.7.14.c8055 21158345

[13] South African National Department of Health. Minimum differentiated models of care package to support linkage to care, adherence and retention in care differentiated models of care standard operating procedures: Adherence Guidelines for HIV, TB and NCDs. Pretoria: South African National Department of Health.

[14] Shigayeva A, Gcwensa N, Ndlovu CD, et al. Retention on ART and viral suppression among patients in alternative models of differentiated HIV service delivery in KwaZulu-Natal, South Africa. PLoS Glob Public Health. 2022;2(12):e0000336. 10.1371/journal.pgph.0000336 36962695 PMC10021436

[15] Mukumbang FC, Ndlovu S, Van Wyk B. Comparing patients’ experiences in three differentiated service delivery models for HIV treatment in South Africa. Qual Health Res. 2022;32(2):238–254. 10.1177/10497323211050371 34911400 PMC8727825

[16] Sharer M, Davis N, Makina N, Duffy M, Eagan S. Differentiated antiretroviral therapy delivery: Implementation barriers and enablers in South Africa. J Assoc Nurs AIDS Care. 2019;30(5):511–520. 10.1097/JNC.0000000000000062 PMC6738628 30720561

[17] Wilkinson L, Harley B, Sharp J, et al. Expansion of the adherence club model for stable antiretroviral therapy patients in the Cape Metro, South Africa 2011–2015. Trop Med Int Health. 2016;21(6):743–749. 10.1111/tmi.12699 27097834

[18] Long L, Kuchukhidze S, Pascoe S, et al. Retention in care and viral suppression in differentiated service delivery models for HIV treatment delivery in sub-Saharan Africa: A rapid systematic review. J Int AIDS Soc. 2020;23(11):e25640. 10.1002/jia2.25640 33247517 PMC7696000

[19] Gobir IB, Niyang MP, Adamu H, et al. Perceived benefits of using smart lockers as virtual dispensing units for chronic disease medication: Healthcare workers’ and patients’ perspective. J Biomed Eng Med Imaging. 2024;11(3):79–96. 10.14738/bjhmr.113.16968

[20] Ntjikelane V, Phiri B, Kaiser JL, et al. Effect of differentiated service delivery models for HIV treatment on healthcare providers’ job satisfaction and workloads in sub-Saharan Africa: A mixed methods study from Malawi, Zambia, and South Africa. Hum Resour Health. 2025;23(1):25. 10.1186/s12960-025-00993-6 40420127 PMC12105310

[21] Bock P, Gunst C, Maschilla L, et al. Retention in care and factors critical for effectively implementing antiretroviral adherence clubs in a rural district in South Africa. J Int AIDS Soc 2019;22(10):e25396. 10.1002/jia2.25396 31588668 PMC6778813

[22] Venables E, Towriss C, Rini Z, et al. Patient experiences of ART adherence clubs in Khayelitsha and Gugulethu, Cape Town, South Africa: A qualitative study. PLoS One. 2019;14(6):e0218340. 10.1371/journal.pone.0218340 31220116 PMC6586296

[23] Muthelo L, Nemagumoni T, Mothiba TM, Phukubje AT, Mabila LN. Experiences of professional nurses regarding the implementation of a central chronic medicine dispensing and distribution program at primary health care facilities in South Africa. Open Public Health J. 2020;13(1):477–483. 10.2174/1874944502013010477

[24] Bogart LM, Shazi Z, MacCarthy S, et al. Implementation of South Africa’s central chronic medicine dispensing and distribution program for HIV treatment: A qualitative evaluation. AIDS Behav. 2022;26(8):2600–2612. 10.1007/s10461-022-03602-y 35122215 PMC8815398

[25] Venter F, Sharif S, Right-e-Pharmacy. Evaluation of the PCU as a CCMDD pick-up point in City of Johannesburg, Phase 2-Adherence Analysis. 2021. Pretoria: DNA Economics; 2021.

[26] Zakumumpa H, Rujumba J, Kwiringira J, Katureebe C, Spicer N. Understanding implementation barriers in the national scale-up of differentiated ART delivery in Uganda. BMC Health Serv Res. 2020;20(1):222. 10.1186/s12913-020-5069-y 32183796 PMC7077133

[27] Luque-Fernandez MA, Van Cutsem G, Goemaere E, et al. Effectiveness of patient adherence groups as a model of care for stable patients on antiretroviral therapy in Khayelitsha, Cape Town, South Africa. PLoS One. 2013;8(2):e56088. 10.1371/journal.pone.0056088 23418518 PMC3571960

[28] Tsondai PR, Wilkinson LS, Grimsrud A, Mdlalo PT, Ullauri A, Boulle A. High rates of retention and viral suppression in the scale-up of antiretroviral therapy adherence clubs in Cape Town, South Africa. J Int AIDS Soc. 2017;20(S4):21649. 10.7448/IAS.20.5.21649 28770595 PMC5577696

[29] Tun W, Apicella L, Casalini C, et al. Community-based antiretroviral therapy (ART) delivery for female sex workers in Tanzania: 6-Month ART initiation and adherence. AIDS Behav. 2019;23:142–152. 10.1007/s10461-019-02549-x 31197700 PMC6773663

[30] Kwarisiima D, Kamya MR, Owaraganise A, et al. High rates of viral suppression in adults and children with high CD4+ counts using a streamlined ART delivery model in the SEARCH trial in rural Uganda and Kenya. J Int AIDS Soc. 2017;20(S4):58–67. 10.7448/IAS.20.5.21673 PMC5577724 28770596

[31] Mokhele I, Huber A, Rosen S, et al. Satisfaction with service delivery among HIV treatment clients enrolled in differentiated and conventional models of care in South Africa: A baseline survey. J Int AIDS Soc. 2024;27:26233. 10.1002/jia2.26233 PMC10963588 38528370

